# Arabidopsis GOLDEN2-LIKE transcription factors interact with ABA-INSENSITIVE 4 to modulate cotyledon greening in light-exposed etiolated seedlings

**DOI:** 10.1093/jxb/erag364

**Published:** 2026-08-04

**Authors:** Pengxin Yu, Friederike Saga, Miriam Bäumers, Ute Hoecker

**Affiliations:** Institute for Plant Sciences and Cluster of Excellence on Plant Sciences (CEPLAS), Biocenter, University of Cologne, Cologne, Germany; Institute for Plant Sciences and Cluster of Excellence on Plant Sciences (CEPLAS), Biocenter, University of Cologne, Cologne, Germany; Center of Advanced Imaging, Heinrich Heine University Düsseldorf, Düsseldorf, Germany; Institute for Plant Sciences and Cluster of Excellence on Plant Sciences (CEPLAS), Biocenter, University of Cologne, Cologne, Germany; University of Glasgow, UK

**Keywords:** Chlorophyll, chloroplast, etiolation, photobleaching, transcription factor

## Abstract

The transcription factors GOLDEN2-LIKE1 (GLK1) and GLK2 are central regulators of chlorophyll biosynthesis and chloroplast biogenesis. We show that GLK1 and GLK2 interact with ABSCISIC ACID INSENSITIVE 4 (ABI4). We reveal that GLKs and ABI4 have antagonistic functions in cotyledon greening of light-exposed etiolated seedlings: compared with the wild type, *abi4* mutants, similar to seedlings overexpressing GLK2, accumulated more protochlorophyllide in dark-grown seedlings, while *glk1 glk2* mutants contained less protochlorophyllide. The protochlorophyllide in etiolated *abi4* mutants and GLK2 overexpressors was inefficiently photoreduced upon light exposure, leading to accumulation of ^1^O_2_. This corresponded to low cotyledon greening rates and low seedling survival. We also identified photosynthesis-associated nuclear genes (*PhANG*s) whose transcript accumulation patterns may contribute to the seedling photobleaching pattern. Importantly, the high protochlorophyllide content, low cotyledon greening rate, high ^1^O_2_ level, and high *PhANG* induction in *abi4* mutant seedlings were fully dependent on *GLK1* and *GLK2*, indicating that *ABI4* acts upstream of *GLK* genes. Since ABI4 did not regulate *GLK* transcript levels while it physically interacted with GLK proteins, these data suggest that ABI4 inhibits GLK1 and GLK2 activities in etiolated seedlings to prevent high protochlorophyllide accumulation which would lead to high ^1^O_2_ levels and seedling death upon light exposure.

## Teaser

The transition from darkness to light is a critical moment that determines seedling survival. We report here that the transcription factor ABI4 inhibits GLK1 and GLK2 activities during seedling etiolation to limit seedling death upon exposure to light.

## Introduction

After seed germination, seedlings are faced with the need to adjust their developmental programs in response to specific light conditions. If seedlings germinate while covered with soil, they will grow in darkness in a process termed etiolation (or skotomorphogenesis), during which specialized chloroplast precursors, known as etioplasts, initiate their development. Within the etioplasts, the enzyme protochlorophyllide reductase (POR) accumulates along with the chlorophyll precursor protochlorophyllide (Pchlide). In angiosperms, PORs are responsible for the only light-dependent step in chlorophyll biosynthesis, and this step occurs in a para-crystalline etioplast compartment called the prolamellar body (PLB), where Pchlide forms a ternary complex with PORs and NADPH. Pchlide in Arabidopsis can be divided into two categories: the photoactive Pchlide is bound by PORs, whereas the non-photoactive Pchlide remains unbound during etiolation (Klein and Schiff, 1972; Lebedev and Timko, 1999; Li *et al*., 2025).

Etiolated seedlings grow upwards through the soil by sensing gravity (Kim *et al*., 2011; Kawamoto and Morita, 2022) and, when they reach the soil surface, they are immediately exposed to light. This is a critical moment that determines plant survival and health, during which the seedlings respond to light in a process termed de-etiolation or photomorphogenesis. Two key photomorphogenic traits are chlorophyll biosynthesis and chloroplast biogenesis (together termed greening), both being crucial for the establishment of photoautotrophic growth. Chlorophyll biosynthesis refers to the enzymatic processes where the PORs convert photoactive Pchlide into chlorophyllide (Chlide) in a reaction termed photoreduction, and Chlide is subsequently converted into chlorophyll (Apel *et al*., 1980; Armstrong *et al*., 1995). In contrast, chloroplast biogenesis is represented by structural changes in the plastid, where etioplasts differentiate into chloroplasts by expanding the thylakoid surface area while integrating and arranging photosynthetic complexes into the thylakoids (Pipitone *et al*., 2021). Chlorophyll is synthesized in Arabidopsis cotyledons within the first 4 h of illumination, while the assembly of a fully photosynthetic chloroplast takes >10 h during de-etiolation (Pipitone *et al*., 2021).

The process of greening requires a balancing act. Both chlorophyll biosynthesis and chloroplast biogenesis are needed for the maximization of the photosynthetic rate. Consequently, having a low chlorophyll content and fewer differentiated chloroplasts can reduce both photosynthetic performance and yield (Kim *et al*., 2023; Frangedakis *et al*., 2024). On the other hand, non-photoactive Pchlide can absorb light energy (termed photosensitization) and transfer this energy to atmospheric oxygen, generating potentially toxic reactive oxygen species (ROS). As a result, excessive Pchlide accumulation during etiolation can lead to seedling lethality by producing ROS during dark-to-light transitions (Meskauskiene *et al*., 2001). Therefore, plastid activity needs to be appropriately repressed in darkness and activated in light for optimized plant development.

Plastid development and activity depend on the transcriptional states of photosynthesis-associated nuclear genes (*PhANG*s), because >90% of chloroplastic proteins are encoded in the nucleus (Martin *et al*., 2002). Multiple classes of transcription factors form a network to tightly regulate the transcription of *PhANG*s. In the dark, PHYTOCHROME-INTERACTING FACTORs (PIFs) act as a major class of *PhANG* repressors (Huq *et al*., 2004; Monte *et al*., 2004; Stephenson *et al*., 2009). PIFs repress etioplast development and Pchlide biosynthesis in concert with ETHYLENE-INSENSITIVE3 (EIN3) and ETHYLENE-INSENSITIVE3-LIKE1 (EIL1) (Zhong *et al*., 2009; Liu *et al*., 2018). In the light, the transcription factors ELONGATED HYPOCOTYL5 (HY5), GATA NITRATE-INDUCIBLE CARBON-METABOLISM-INVOLVED (GNC), CYTOKININ-RESPONSIVE GATA1 (CGA1), MYBs, and GOLDEN2-LIKE (GLK) promote greening by activating *PhANG* transcription either alone or cooperatively (Oyama *et al*., 1997; Fitter *et al*., 2002; Chiang *et al*., 2012; Frangedakis *et al*., 2024; Zhang *et al*., 2024).

GOLDEN2-LIKE 1 and 2 (GLK1 and 2) are key greening regulators that have been used in significant agricultural applications, where ectopic expression of maize GLKs improved yield in rice by 30–70% (Li *et al*., 2020; Yeh *et al*., 2022). GLKs exist in most plants as a paralogous pair (Rossini *et al*., 2001; Fitter *et al*., 2002; Powell *et al*., 2012; Tu *et al*., 2022), and they mainly act in the nuclei of mesophyll cells (Waters *et al*., 2008), where they promote the transcription of the *PhANG*s encoding thylakoid-associated protein components and chlorophyll biosynthetic enzymes (Waters *et al*., 2009; Tu *et al*., 2022). The necessity of fine-tuning GLK activity was demonstrated by M. Li *et al*. (2022), who reported that two transcription factors physically and antagonistically interacted with GLKs, ultimately modulating cell death in response to external stress. Although *GLK* transcript levels are low in darkness (Fitter *et al*., 2002), and GLK protein is degraded in darkness (D. Zhang *et al*., 2021), GLKs still play a physiological role in seedling etiolation. Specifically, Arabidopsis *glk1 glk2* mutant seedlings accumulate less Pchlide and contain smaller etioplasts with smaller PLBs when compared with the wild type (Waters *et al*., 2009). Therefore, maintaining a moderate level of GLK activity during etiolation may also be vital for seedling health. Since multiple studies have demonstrated the physiological significance of the post-translational regulation of GLKs in different plant species (Rauf *et al*., 2013; Tang *et al*., 2016; Ni *et al*., 2017; C. Zhang *et al*., 2021; D. Zhang *et al*., 2021, Y. Li *et al*., 2022; Han *et al*., 2024; Tachibana *et al*., 2024; Zhang *et al*., 2024; Sun *et al*., 2025), we screened for other transcription factors that physically interact with GLK1 or GLK2 from Arabidopsis.

## Materials and methods

### Plant material and growth conditions

All Arabidopsis lines used in this study were in the Columbia ecotype, and we list their NASC stock IDs here. Both the *glk1 glk2* mutant (N9807) and the *35Spro*::*GLK2*-*GFP* line (N2107721) were described previously (Fitter *et al*., 2002; Waters *et al*., 2008). The mutants *abi4-1* (N73626), *gl1* (N1688), *abi4-103 gl1* (N3838), and *abi4-t* (N580095) are described in the text.

Seeds were surface-sterilized, stratified for 3 d in darkness at 4 °C, then sown on Murashige and Skoog (MS) medium containing 10 g l^–1^ agar (SERVA) (without sucrose unless indicated otherwise). The sown seeds were exposed to white light (100 μmol m^–2^ s^−1^) for 4 h to induce germination, then left in a growth chamber either under the same white light or in darkness at 21 °C. For analysis of chlorophyll content, plants were grown on soil in a climate-controlled walk-in growth chamber under a long-day photoperiod (16 h of 100 μmol m^–2^ s^−1^ white light/8 h darkness) at 21 °C.

### Generation and verification of the *glk1 glk2 abi4-1* mutant

The *glk1 glk2 abi4-1* triple mutant was generated by crossing *glk1 glk2* with *abi4-1*. From the F_2_ population, those homozygous for *glk1* and *glk2* were identified by their pale-green cotyledons. Genomic DNA was isolated individually from these plants and subjected to a dCAPS (derived cleaved amplified polymorphic sequence) analysis for *abi4-1* homozygosity. A sequence encompassing the *abi4-1* locus was PCR-amplified, and the PCR products were digested by *Alu*I. The sequences of primers used for dCAPS analysis are listed in Supplementary Table S1.

### Germination assay

Surface-sterilized and stratified seeds were sown on MS agar plates with or without 1.5 μM abscisic acid (ABA). The plates were placed directly into the growth chamber under the long-day photoperiod (16 h of 100 μmol m^−2^ s^−1^ white light/8 h darkness) at 21 °C. After 7 d, the seeds were assessed for germination based on radicle protrusion. Three separate plates derived from the same seed batch served as replicates.

### Cloning of constructs

For yeast two-hybrid (Y2H) assays, we generated the entry clones for BD-GLK1_Δ1–154_, BD-GLK2_Δ1–148_, BD-ABI4_Δ188–289_, AD-GLK1, and AD-GLK2 by first PCR-amplifying the coding sequences from Arabidopsis cDNA and cloning them into pENTR3C (Thermo Fisher) via homologous recombination (ClonExpress, Vazyme). The entry clone for AD-ABI4 was obtained by isolating the pDEST22-ABI4 vector from the REGIA Y2H library (Paz-Ares and Regia Consortium, 2002) and mixing pDEST22-ABI4 with pDONR221 (Thermo Fisher) in a BP Gateway reaction (Thermo Fisher). Then the destination vectors were each cloned by mixing the respective entry clone with a GAL4-based BD or AD vector (pAS2-GW and pACT2-GW) (Clontech) in an LR Gateway reaction (Thermo Fisher).

For FRET–FLIM (Förster resonance energy transfer–fluorescence lifetime imaging microscopy) assays, pAMARENA (Steffens *et al*., 2014) and pENSG-YFP (Laubinger *et al*., 2006) were used for N-terminal tagging with mCherry and yellow fluorescent protein (YFP), respectively. The destination vectors were cloned via LR reactions using either the entry clone pENTR3C-GLK or pDONR221-ABI4.

For the luciferase complementation imaging (LCI) assay, all nLUC and cLUC expression vectors were generated by conventional digestion–ligation cloning. GLK1 and GLK2 were cloned into the pCambia1300-nLUC vector, and ABI4 was cloned into pCambia1300-cLUC (Chen *et al*., 2008), in all cases using the *Kpn*I and *Sal*I restriction sites. The sequences of primers used for cloning are listed in Supplementary Table S1.

### Yeast two-hybrid assays

Gal4-based Y2H assays, specifically the histidine growth selection assay, were performed. The BD- and AD-plasmids were transformed into the yeast strain AH109 (Clontech) using the Frozen-EZ Yeast Transformation II Kit (Zymo Research). Transformants were then selected on synthetic drop-out auxotrophic plates (SD-L-W) (Takara Bio) containing 2% glucose. Ten co-transformed yeast colonies were suspended in sterile water and plated on both SD-L-W and SD-L-W-H plates containing 2% glucose. The yeast was grown at 30 °C, and 3-aminotriazole (3-AT) was used in the SD-L-W-H plates to prevent autoactivation.

### Förster resonance energy transfer–fluorescence lifetime imaging microscopy assays

For the FRET–FLIM assays, leek epidermal cells were transiently transformed with different FRET constructs by particle bombardment as described by Ponnu *et al*. (2019). The FLIM experiments were also performed according to Ponnu *et al*. (2019), with the difference that the curve-fitting processes were done in the software SymPhoTime 64 (PicoQuant).

### Firefly luciferase complementation imaging assay

Constructs expressing GLK1, GLK2, or ABI4 proteins fused to the C- or N-terminal half of firefly luciferase were transformed into the *Agrobacterium tumefaciens* strain *GV3101::pMP90*, and different combinations of transformed *Agrobacterium* were infiltrated into tobacco leaves. The infiltration procedure and luminescence imaging were performed as described in Kreiss *et al*. (2023). The luminescence intensity was quantified, and the bright-field and luminescence images were overlapped using Fiji (Schindelin *et al*., 2012). Data were plotted without normalization.

### Protochlorophyllide, chlorophyllide, and chlorophyll quantification

For Pchlide and Chlide quantification, 15 etiolated seedlings per replicate were harvested from MS agar plates in darkness. The samples were lysed and homogenized in 300 μl of ice-cold 80% acetone, then centrifuged at 5000 *g* for 2 min, and 100 μl of the supernatant was aliquoted into a white 96-well plate. The fluorescence emission spectra of the sample were scanned from 600 nm to 700 nm using a spectrophotometer with an excitation wavelength of 440 nm (Tecan Infinite 200 PRO).

For chlorophyll quantification of plants grown in a long-day photoperiod, 50–100 mg of leaf tissue per replicate was harvested and weighed, frozen in liquid nitrogen, and ground to fine powder with a TissueLyser (Qiagen). A 1.5 ml aliquot of 80% acetone was added and samples were vortexed for 5 min, then centrifuged at 9740 *g* for 5 min. Finally, 100 μl of the supernatant was aliquoted into a transparent 96-well plate, and the absorbance was measured at 645 nm and 663 nm (Tecan Infinite 200 PRO). Total chlorophyll in mg gFW is calculated using the following formula: [(*A*_663_×0.00802)+(*A*_645_×0.0202)]×1.5/FW (Arnon, 1949). For chlorophyll quantification in the cotyledon greening assay, the procedure was kept almost the same, with the difference that 25 representative seedlings per replicate were harvested, frozen, ground, then added to 150 µl of 80% acetone. Total chlorophyll in ng per seedling is calculated using the following formula: [(*A*_663_×8.02)+(*A*_645_×20.2)]×0.15/25.

### Cotyledon greening assays

Around 100 seeds sown on each replicate MS agar plate were stratified, grown for varied numbers of days in the dark, followed by 3 d in constant white light (Wc; 100 μmol m^−2^ s^−1^), all at 21 °C. After 3 d, the germinated seedlings were classified into green/bleached by manually examining the phenotype of individual seedlings.

### Fluorescence imaging of Singlet Oxygen Sensor Green staining

Seedlings were incubated with 100 μM Singlet Oxygen Sensor Green (SOSG; Lumiprobe) in the dark for 20 min. The excess SOSG solution was then removed, and the seedlings were washed with sterile water. The SOSG signal was captured by laser excitation at 488 nm and emission at 525 nm. Chlorophyll autofluorescence was visualized by excitation at 610 nm and emission at 630 nm. The laser and detector settings were kept the same for all measurements. The imaging was performed using the Leica TCS SP8 (Leica microsystems). Image analyses were performed in Fiji (Schindelin *et al*., 2012).

### Reverse transcription–quantitative PCR

Total RNA was extracted from Arabidopsis seedlings (∼40 seedlings per replicate), using the NucleoSpin RNA Plant Mini Kit (Macherey Nagel) according to the manufacturer’s instructions. For cDNA synthesis, 1 µg of RNA, 2 µl of 10 µM Oligo-dT primer, and RNase-free water were added to a total volume of 27 µl. The reaction was incubated at 65 °C for 5 min, then cooled on ice for 2 min. Subsequently, 8 µl of 5× RevertAid buffer, 4 µl of 10 mM dNTPs, and 1 µl of RevertAid H Minus Reverse Transcriptase (Thermo Fisher) were added to the reaction, and the mix was incubated at 37 °C for 5 min, at 42 °C for 1 h for cDNA synthesis, then at 70 °C for 5 min for heat inactivation.

For reverse transcription–quantitative PCR (RT–qPCR) analysis, 0.5 µl of cDNA was mixed with 0.5 µl of 10 µM forward and reverse primers and 5 µl of GoTaq qPCR Master Mix (Promega) in a 10 µl reaction. The RT–qPCR was performed on a QuantStudio 5 Real-Time PCR System (Thermo Fisher). The relative transcript levels were calculated using the 2^−ΔΔCt^ method (Livak and Schmittgen, 2001), and the transcript fold change was normalized to that of the housekeeping gene *ACT2*. The Ct value for *ACT2* remained stable between different experimental groups (Supplementary Fig. S1). At least four biological replicates were used in each experiment. The sequences of primers used for qPCR are listed in Supplementary Table S1.

### Transmission electron microscopy

Seeds sown on MS agar plates were first stratified, then kept in darkness at 21 °C for 5 d. Next, cotyledon sections were prepared over a five-day period. On Day 1, whole seedlings (∼30 per genotype) were fixed in 2.5% glutaraldehyde+4% paraformaldehyde in 0.1 M phosphate buffer (pH 7.4) by vacuum infiltration for 1 h under a fume hood and stored overnight at 4 °C. On Day 2, samples were washed twice quickly and then three times for 5 min each with 0.1 M phosphate buffer (pH 7.4) at room temperature. Post-fixation was carried out in osmium solution (1% OsO_4_, 2 mM CaCl_2_, 0.8% potassium ferricyanide in 0.1 M phosphate buffer pH 7.4) for 1 h in the dark on ice. Next, samples were washed seven times with 0.1 M phosphate buffer (pH 7.4) at room temperature. Seedlings were then dehydrated in 50% ethanol (1 h on ice), and subsequently in 70% ethanol (overnight at 4 °C). On Day 3, *en bloc* staining was performed by incubating samples in 1% uranyl acetate in 70% ethanol for 1 h in the dark at room temperature, then seedlings were washed twice quickly and then five times for 5 min each in 70% ethanol on ice. Dehydration was carried out by a graded ethanol series (80, 90, 96, and 100%) for 30 min at each step (100% twice) on ice. Afterwards, the samples were washed with 100% acetone (2×30 min), followed by overnight rotation in an Epon™:acetone (1:3) mixture (Merck, Epoxy Embedding Medium kit, cat. no. 45359-1EA-F) at room temperature. On Day 4, Epon™ infiltration was continued by rotating samples in progressively higher concentrations of Epon™:acetone (1:2 for 30 min, 1:1 for 1 h, 2:1 for 1 h, and 3:1 for 2×1 h), followed by incubation in pure Epon™ for 2 h. Fresh Epon™ was then added for the overnight rotation at room temperature. On Day 5, seedlings were incubated in fresh Epon™ for 2 h at room temperature, then the cotyledons were excised and embedded in molds filled with Epon™, which were polymerized at 65 °C for 72 h. Ultrathin sections (70 nm) were prepared using an ultramicrotome (UC Enuity, Leica Microsystems). Sections were stained with 2% aqueous uranyl acetate for 5 min, rinsed with distilled water, and subsequently stained with Reynolds’ lead citrate (Reynolds, 1963) for 4 min under CO_2_-free conditions, followed by a final rinse with distilled water. The sections were examined with a transmission electron microscope (Zeiss EM 902) operated at 80 keV, and images were captured using a slow-scan CCD camera (Type 7899). Three seedlings per genotype were examined.

### Statistical analyses

All statistical analyses were performed in the R environment (R Core Team, 2019), and the data were visualized using the ggplot2 package (Wickham, 2009). Each individual replicate is plotted as a dot, and summary statistics are plotted as indicated in each figure. For boxplots, the hinges on each box indicate the lower and upper quantiles, and the whiskers show the data range. The median is represented by a horizontal line within the box. No data were excluded from the analyses.

### Accession numbers

The Arabidopsis genes included in this study can be identified in The Arabidopsis Information Resource (www.arabidopsis.org) under these accession numbers: *ABI4* (AT2G40220), *GLK1* (AT2G20570), *GLK2* (AT5G44190), *PORA* (AT5G54190), *PORB* (AT4G27440), *HEMA1* (AT1G58290), *CHLH* (AT5G13630), *GUN4* (AT3G59400), *LHCB2*.1 (AT2G05100), *LHCB2*.*2* (AT2G05070), *LHCB2*.*3* (AT3G27690), *LHCB3* (AT5G54270), and *LHCB6* (AT1G15820).

## Results

### ABI4 and GLKs interact in yeast two-hybrid assay and *in planta*

To identify proteins interacting with GLKs, we screened an Arabidopsis transcription factor library (Paz-Ares and Regia Consortium, 2002) using a Y2H workflow devised by Castrillo *et al*. (2011). In order to use GLKs as bait, we first deleted the transactivation domain from the GLKs, then performed the screen using the GLK C-terminal domains as baits (Fig. 1A). We identified an interaction between ABI4 and the C-terminal domain of GLK2 (Fig. 1B).

**Fig. 1.**
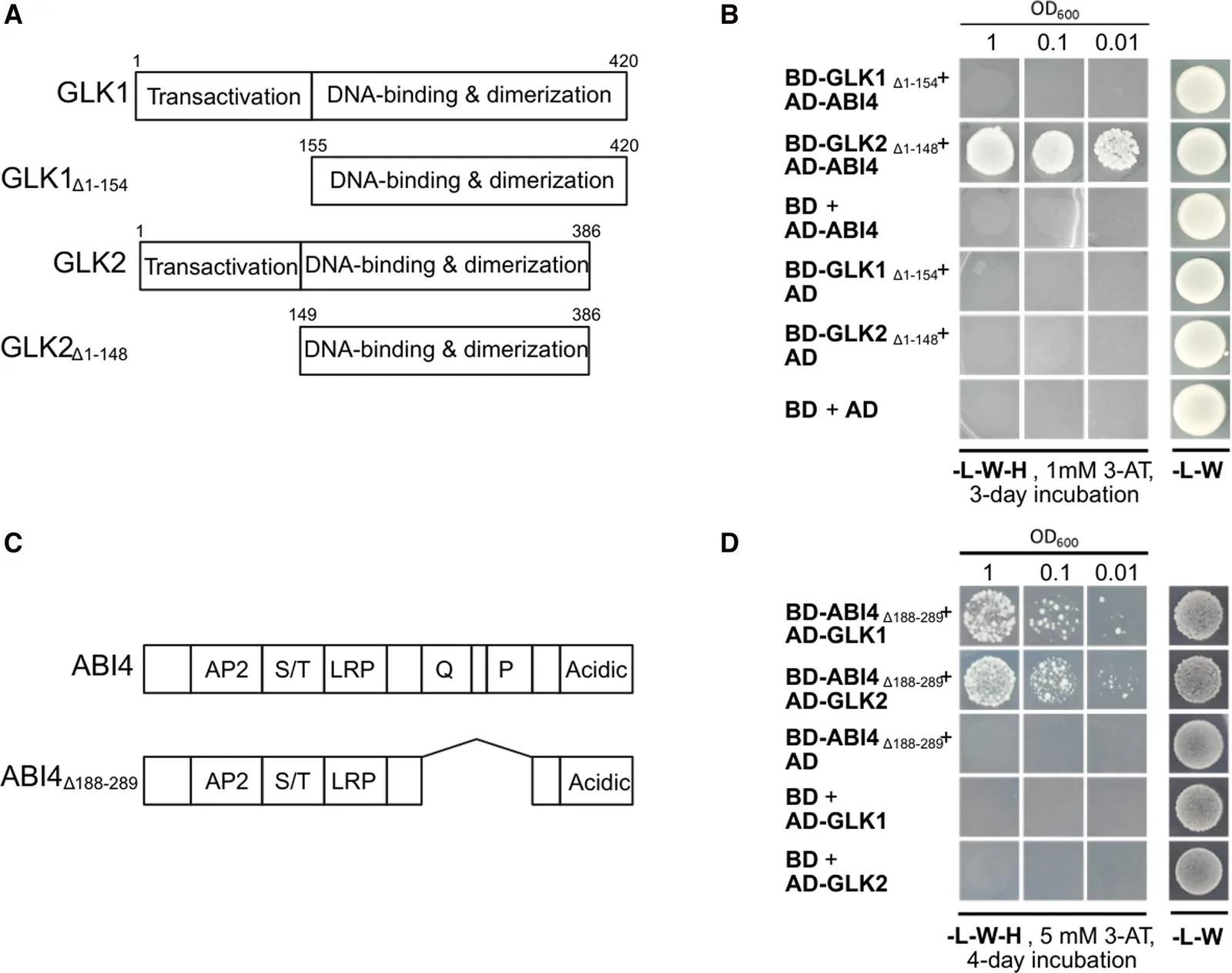
GLK1 and GLK2 physically interact with ABI4 in yeast two-hybrid assay. (A) Scheme of GLK1 and GLK2 proteins and deletion derivatives lacking the transactivation domain. The latter were used as bait in the Y2H screen. (B) GLK2_Δ1–148_ interacts with ABI4. (C) Scheme of ABI4 protein and a deletion derivative lacking the transactivation domain; the latter was used as the bait in the following Y2H assay. AP2, APETALA2 domain; S/T, serine-/threonine-rich domain; LRP, LRP motif; Q and P, glutamine- and proline-rich domains, respectively. (D) ABI4_Δ188–289_ interacts with full-length GLK1 and GLK2.

GLK1 and GLK2 share conserved regions and functional redundancy (Waters *et al*., 2009; Rauf *et al*., 2013). Since previous reports showed that proteins often interact with both GLK1 and GLK2 (Zhang *et al*., 2024; Sun *et al*., 2025; Tachibana *et al*., 2025), we hypothesized that ABI4 interacts with GLK1 as well as GLK2. Although the GLK1 C-terminal domain did not interact with ABI4 (Fig. 1B), we hypothesized that GLK1 N-terminal domains may be necessary for an interaction between GLK1 and ABI4. To test this, we deleted the transactivating glutamine-rich and proline-rich domains from ABI4 so that we could use ABI4 as a bait (Fig. 1C). Indeed, ABI4_Δ188–289_ interacted with full-length GLK1 and GLK2 in Y2H assay (Fig. 1D).

We further confirmed the ABI4–GLK interaction *in planta*. First, we performed FRET–FLIM experiments after co-expressing YFP-tagged ABI4 and mCherry-tagged GLK proteins in leek epidermal cells by particle bombardment. As shown in Fig. 2A and B, the lifetime of the donor fluorophore YFP was significantly reduced when YFP–ABI4 was co-expressed with either mCherry–GLK1 or mCherry–GLK2 compared with co-expression with mCherry, supporting an interaction between Arabidopsis ABI4 and GLK1 as well as GLK2. Next, we performed an LCI assay after co-expressing nLUC- and cLUC-fused proteins in tobacco by *Agrobacterium* infiltration. We observed that both GLK1–nLUC and GLK2–nLUC generated luminescence when co-expressed with cLUC–ABI4 (Fig. 2C, D), whereas the negative controls did not generate luminescence, supporting an interaction between Arabidopsis ABI4 and GLK in tobacco. Together, these assays corroborate the existence of an *in planta* interaction between ABI4 and both GLKs.

**Fig. 2.**
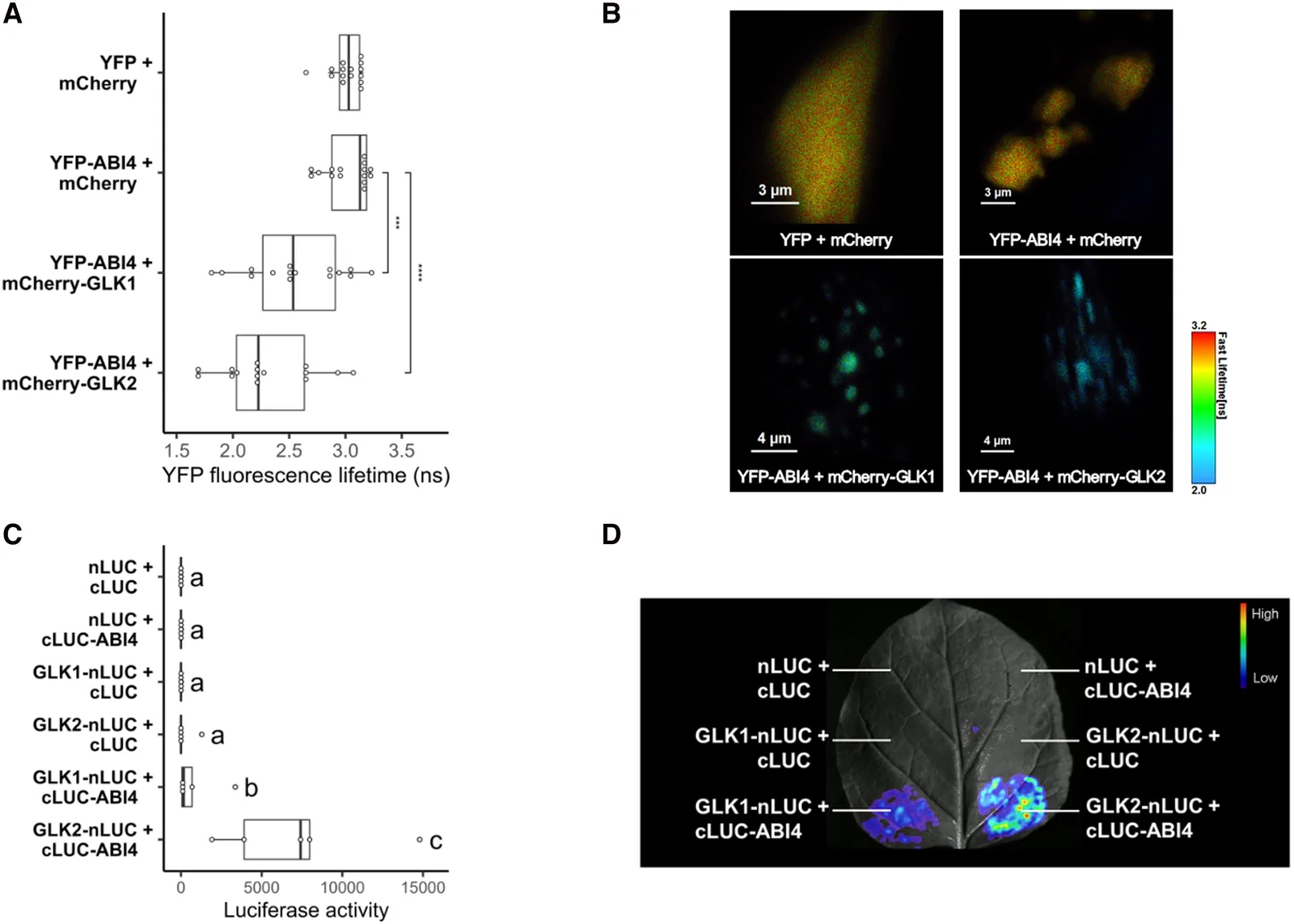
GLK1 and GLK2 physically interact with ABI4 *in planta*. (A) Lifetime of the donor fluorophore (YFP) measured by FRET–FLIM inside the nuclei of leek epidermal cells after particle bombardment, *n*=15 nuclei. Asterisks indicate a significant difference in lifetime between the indicated pair (Student’s *t*-test, ***P<0.001, ****P<0.0001). (B) FLIM images of the representative nuclei from (A), showing changes in YFP lifetime. (C) Quantification of luminescence detected by a CCD camera from a luciferase complementation imaging assay in tobacco leaves, *n*=5 leaves. Combinations which are significantly different (*P*<0.05) are labeled with different letters (pairwise Wilcoxon test with multiple testing correction). (D) Representative leaf image from (C) showing the luminescence signal in sectors transfected with the indicated plasmid combinations.

### Etiolated *abi4* mutant seedlings display *GLK*-dependent protochlorophyllide overaccumulation and inefficient protochlorophyllide photoreduction

The ABI4–GLK interaction prompted us to investigate the biological relevance of this interaction. To this end, we compared the phenotypes of *abi4* and *glk1 glk2* loss-of-function mutants. First, because GLKs promote chlorophyll accumulation in light (Fitter *et al*., 2002), we quantified the chlorophyll content in seedlings grown on soil in a long-day photoperiod for 10 d. As described previously (Waters *et al*., 2008), the *glk1 glk2* double mutant accumulated significantly less chlorophyll than the Col-0 wild type, while the *35Spro*::*GLK2-GFP* line accumulated slightly more chlorophyll than the wild type, though the difference was not statistically significant (Supplementary Fig. S2). We included two *abi4* knockout mutant alleles: *abi4-1*, with a 1 bp deletion at *ABI4* codon 157 in the Col-0 background (Finkelstein *et al*., 1998); and *abi4-103 gl1*, with a nonsense point mutation at *ABI4* codon 39 in the *gl1* background (Laby *et al*., 2000). Both *abi4* mutants accumulated similar levels of chlorophyll compared with their wild-type genotypes, indicating that the ABI4–GLK interaction does not play a role in chlorophyll homeostasis under these conditions.

Next, we hypothesized that the ABI4–GLK interaction plays a specific role in Pchlide accumulation during seedling skotomorphogenesis. In the following phenotypic assays, we included two *abi4* knockout mutant alleles: the above-mentioned *abi4-1*, and *abi4-t* which contains a T-DNA insertion in *ABI4* (Alonso *et al*., 2003; Shu *et al*., 2013). To elucidate the genetic relationships between *ABI4* and *GLK* genes, we generated the *glk1 glk2 abi4-1* triple mutant by crossing *glk1 glk2* with *abi4-1*. The *abi4-1* homozygosity in the *glk1 glk2 abi4-1* triple mutant was verified by genotyping using a dCAPS marker (Supplementary Fig. S3A) and observing a complete insensitivity to 1.5 µM ABA during seed germination (Supplementary Fig. S3B). The *glk1 glk2* homozygosity in the *glk1 glk2 abi4-1* triple mutant was confirmed by the observation that the triple mutant has the same chlorophyll-deficient phenotype as the *glk1 glk2* double mutant (Fig. 4A).

To determine Pchlide levels, we extracted and quantified the total Pchlide content in 5-day-old etiolated seedlings. We found that in comparison with the wild type, the *glk1 glk2* mutant accumulated significantly less Pchlide, while the *35Spro*::*GLK2*-*GFP* line accumulated more Pchlide (Fig. 3A, B; Supplementary Fig. S4A). Importantly, both *abi4* mutants had higher levels of Pchlide, thus resembling the *35Spro*::*GLK2*-*GFP* line, while the Pchlide content returned to a wild-type level in the *glk1 glk2 abi4-1* mutant (Fig. 3A, B; Supplementary Fig. S4A). This revealed that the total Pchlide overaccumulation in etiolated *abi4* mutants was completely dependent on *GLK* genes. We noticed that Pchlide levels in the *glk1 glk2 abi4* triple mutant were slightly higher than in the *glk1 glk2* double mutant—though not statistically significant. Thus, it is possible that a *GLK*-independent mechanism may provide a small contribution to the *abi4* mutant phenotype. Alternatively, residual *GLK1* transcript present in the *glk1* mutant (Fitter *et al*., 2002) may be responsible.

**Fig. 3.**
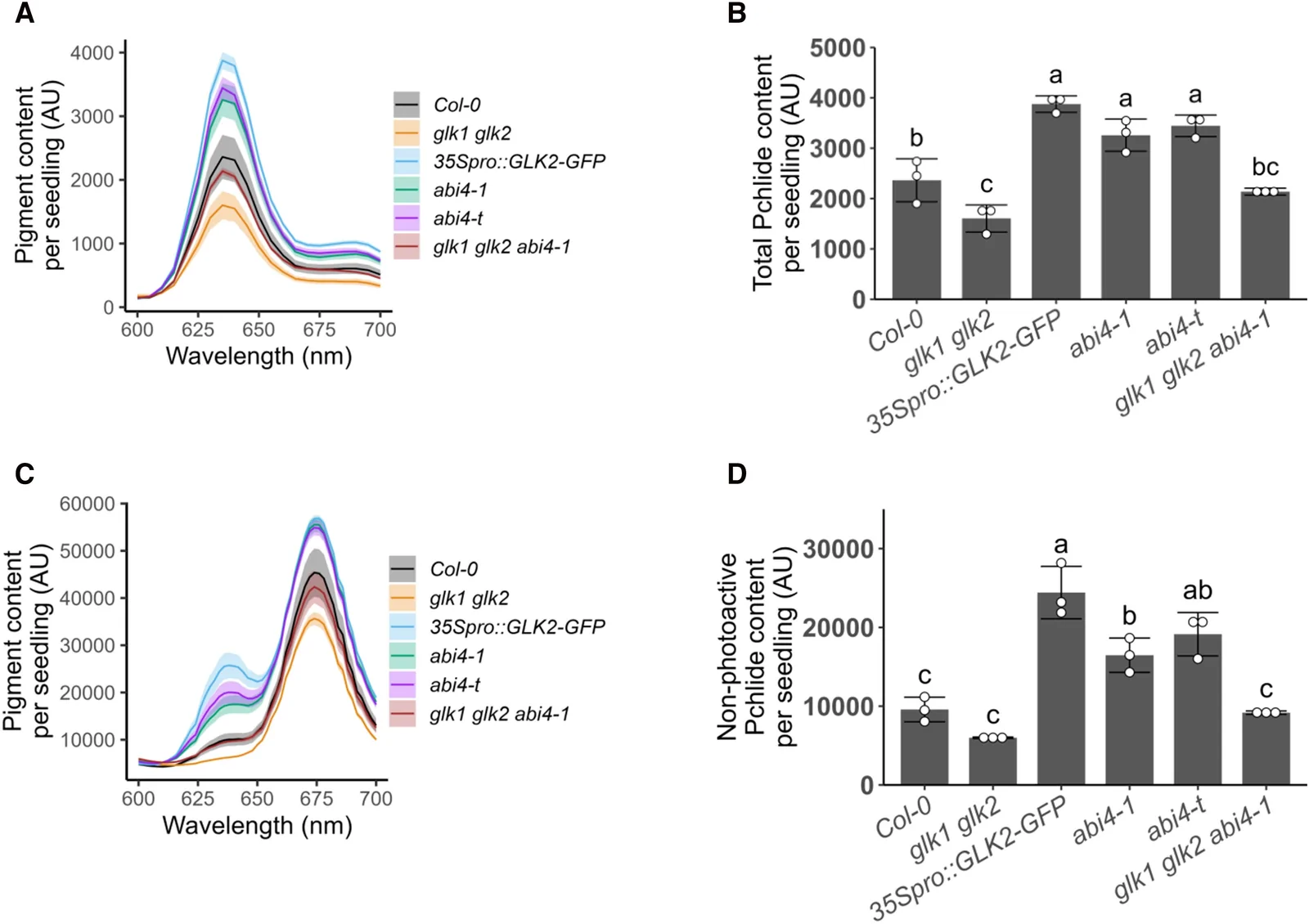
Etiolated *abi4* mutants overaccumulate Pchlide and cannot efficiently convert all Pchlide into Chlide, and these phenotypes are dependent on *GLK1* and *GLK2*. (A) Fluorescence of Pchlide at different emission wavelengths in 5-day-old etiolated seedlings; data are plotted as the mean (solid lines) ±SD (shaded areas). (B) Comparison of total Pchlide content by the peak fluorescence at 634 nm from (A). Genotypes which are significantly different are labeled with different letters (*n*=3 replicate extracts, ANOVA and Tukey’s post-hoc tests); error bars indicate the SD. (C) Fluorescence of Pchlide (634 nm) and Chlide (670 nm) in 5-day-old etiolated seedlings after transfer from darkness to 100 µmol m^−2^ s^−1^ white light for 5 min. (D) Comparison of non-photoactive Pchlide content using the peak fluorescence at 634 nm from (C). Genotypes which are significantly different are labeled with different letters (*n*=3 replicate extracts, ANOVA and Tukey’s post-hoc tests); error bars indicate the SD.

According to previous studies, photoactive Pchlide is converted by PORs to Chlide by 1 ms of white light, while non-photoactive Pchlide is not efficiently photoreduced (Shibata, 1957; Xu *et al*., 2016). Therefore, we measured the non-photoactive Pchlide levels in 5-day-old etiolated seedlings exposed to 5 min of white light. We found that, similar to the *35Spro*::*GLK2*-*GFP* line, neither *abi4* mutant efficiently converted all Pchlide to Chlide (Fig. 3C), because they still contained more non-photoactive Pchlide than the wild type upon light exposure (Fig. 3D; Supplementary Fig. S4B). In contrast, all Pchlide was photoreduced in both the *glk1 glk2* and the *glk1 glk2 abi4-1* mutant (Fig. 3C), where the non-photoactive Pchlide content returned to a wild-type level (Fig. 3D; Supplementary Fig. S4B). We conclude that etiolated *abi4* mutant seedlings display inefficient Pchlide photoreduction and non-photoactive Pchlide overaccumulation, and that these phenotypes are completely dependent on *GLK* genes.

### Etiolated *abi4* mutant seedlings exhibit a *GLK*-dependent reduced greening rate when exposed to light

Non-photoactive Pchlide is a photosensitizer that can cause pigment photobleaching and inhibition of greening (Meskauskiene *et al*., 2001; Erdei *et al*., 2005). Since etiolated *abi4* mutant seedlings contained more non-photoactive Pchlide compared with the wild type, we performed cotyledon greening assays using seedlings that were first etiolated for 1–5 d and then exposed to 3 d of white light. A visual examination revealed that 2-day-old etiolated seedlings of all investigated genotypes developed green cotyledons after transfer to white light (Fig. 4A, first row). However, when seedlings were etiolated for 5 d before the transfer to white light (Fig. 4A, second row), a small portion of the Col-0 wild-type seedlings failed to develop green cotyledons. In comparison, most seedlings of the *35Spro*::*GLK2-GFP* line and the two *abi4* mutants had cotyledons that failed to green, and this low greening rate was rescued in the *glk1 glk2 abi4*-*1* triple mutant. After prolonging the light exposure to 10 d (Fig. 4A, third row), we observed that higher cotyledon greening rates corresponded to higher seedling establishment and survival rates, while seedlings that failed to green showed arrested development. We therefore designate the fail-to-green cotyledon phenotype as a photobleaching phenotype. Since sucrose is known to suppress cotyledon photobleaching by stimulating Pchlide photoreduction (Barnes *et al*., 1996; Mu *et al*., 2022), we assessed the effect of adding 2% sucrose exogenously to the culture medium. As shown in Fig. 4A (fourth row), sucrose indeed partially rescued cotyledon photobleaching, especially in the *35Spro*::*GLK2-GFP* line and the two *abi4* mutants, suggesting that the photobleaching phenotype in these lines could be due to inefficient Pchlide photoreduction.

**Fig. 4.**
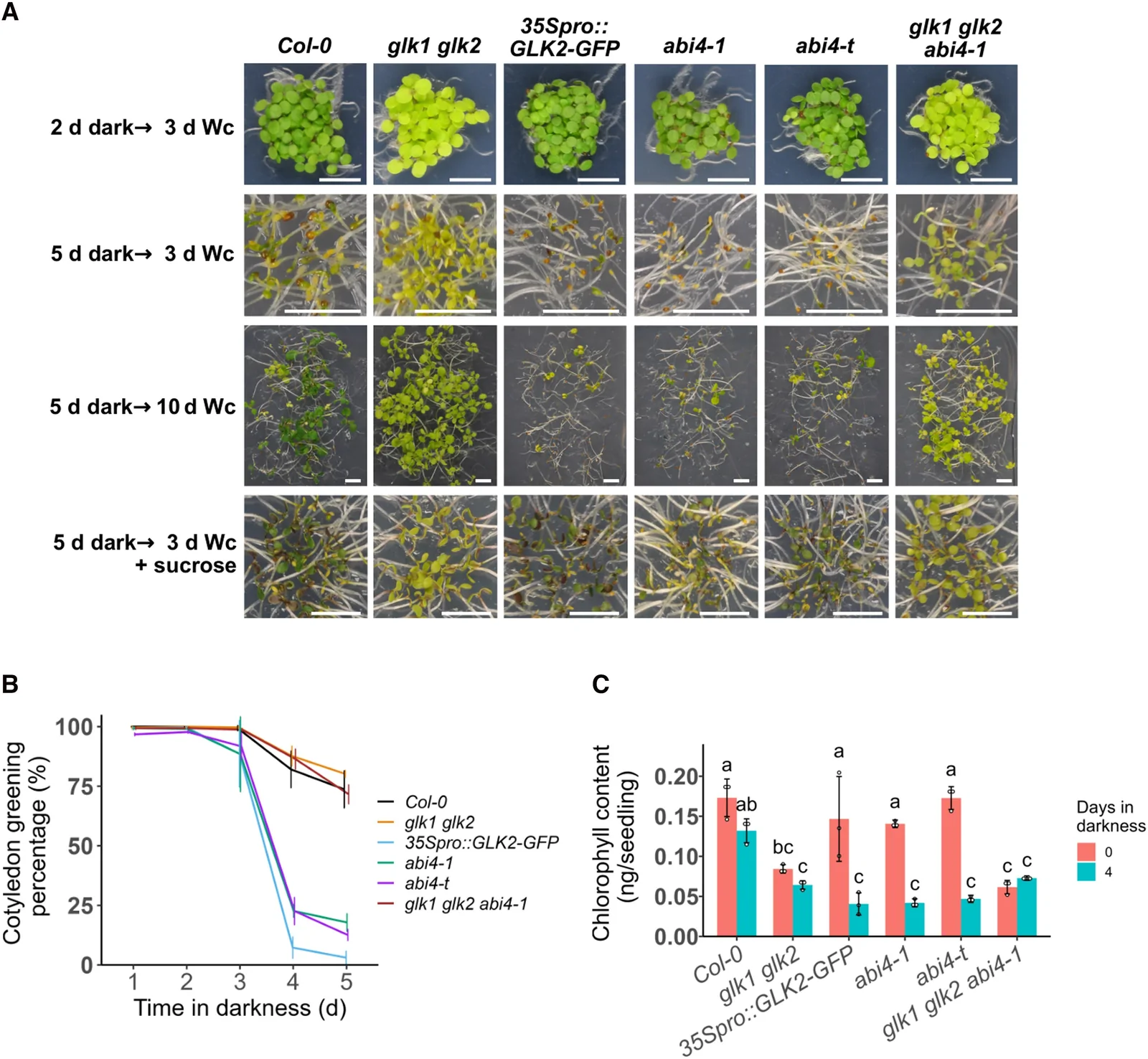
A *35Spro*::*GLK2*-*GFP* line and two *abi4* mutants fail to green when seedlings etiolated for >4 d are exposed to light. (A) Photographs of seedlings that were etiolated for the indicated number of days followed by transfer to constant white light (Wc) for the indicated time. Seedlings shown in the last row were grown on 2% sucrose. Scale bars=0.5 cm. (B) Quantification of cotyledon greening percentage of etiolated seedlings exposed to Wc for 3 d, *n*=3 replicate plates; error bars indicate the SD. (C) Quantification of chlorophyll content of seedlings grown either without prior etiolation or with 4 d of etiolation, then exposed to Wc for 5 d. Genotypes which are significantly different are labeled with different letters (*n*=3 replicate extracts, ANOVA and Tukey’s post-hoc tests); error bars indicate the SD.

We quantified the proportion of greened seedlings by visually categorizing seedlings as green or bleached. As seen in Fig. 4B, the *35Spro*::*GLK2-GFP* line and the two *abi4* mutants displayed consistently lower greening rates than Col-0 and *glk1 glk2* when etiolation lasted longer than 3 d. Importantly, the *glk1 glk2* mutations in the *abi4*-*1* background rescued the failure of *abi4-1* to green. To further quantify the degree of photobleaching, we measured the chlorophyll levels in seedlings grown either without etiolation or with 4 d of etiolation prior to exposure to white light for 5 d. As shown in Fig. 4C, in seedlings grown without prior etiolation, Col-0, *35Spro*::*GLK2-GFP*, and the two *abi4* mutants contained similar levels of chlorophyll, whereas the chlorophyll levels of *glk1 glk2* and *glk1 glk2 abi4*-*1* mutants were similar to each other, but significantly lower than that of the wild type. The 4 d of prior etiolation did not significantly change the light-induced chlorophyll accumulation level in Col-0 or *glk1 glk2* mutant seedlings, but it significantly decreased the seedling chlorophyll levels in *35Spro*::*GLK2-GFP* and the two *abi4* mutants. Strikingly, the *glk1 glk2* mutations rescued the inhibited chlorophyll accumulation caused by the 4 d of prior etiolation in the *abi4-1* mutant background, demonstrating again that the *GLK* genes are necessary for the photobleaching of light-exposed etiolated seedlings. To summarize, etiolated *abi4* mutants and *35Spro*::*GLK2-GFP* seedlings have lower greening rates compared with the wild type when exposed to light, and this photobleaching phenotype is dependent on *GLK* genes.

### Photobleaching of the etiolated seedlings corresponds to singlet oxygen accumulation in the cotyledons

Although multiple species of ROS exist in Arabidopsis, the cause of Pchlide-induced photobleaching has been attributed to the generation of singlet oxygen (^1^O_2_) during photosensitization (Meskauskiene *et al*., 2001; Triantaphylidès *et al*., 2008). Hence, we quantified the ^1^O_2_ generated in 5-day-old etiolated seedlings before and after exposure to light. To detect ^1^O_2_  *in planta*, we stained seedlings with the fluorescent probe SOSG (Flors *et al*., 2006) and then detected both SOSG fluorescence and chlorophyll autofluorescence in the cotyledons using a confocal microscope. Before exposure to white light (0 d Wc), all genotypes contained a low level of ^1^O_2_ and displayed limited chlorophyll autofluorescence (Fig. 5A, B). After 3 d of light exposure (3 d Wc), the *35Spro::GLK2-GFP* line and both *abi4* mutants failed to accumulate chlorophyll, and this phenotype correlated with high ^1^O_2_ accumulation levels. In contrast, light-exposed wild-type seedlings and—to a lesser extent—*glk1 glk2* and *glk1 glk2 abi4*-*1* mutant seedlings exhibited chlorophyll autofluorescence and low levels of ^1^O_2_. Together, these ^1^O_2_ levels are consistent with the Pchlide and photobleaching phenotypes reported above: when compared with the wild type, etiolated *abi4* mutant and *35Spro::GLK2-GFP* seedlings contained higher levels of non-photoactive Pchlide and displayed low greening rates and high ^1^O_2_ content when exposed to light. Again, the lower ^1^O_2_ level generated by photosensitization in the *glk1 glk2 abi4-1* mutant seedlings compared with that in the *abi4-1* mutant seedlings supports the conclusion that the photobleaching observed in etiolated *abi4* mutant seedlings is dependent on GLK activity, and that GLKs regulate Pchlide accumulation to control subsequent ^1^O_2_ production when etiolated seedlings are exposed to light.

**Fig. 5.**
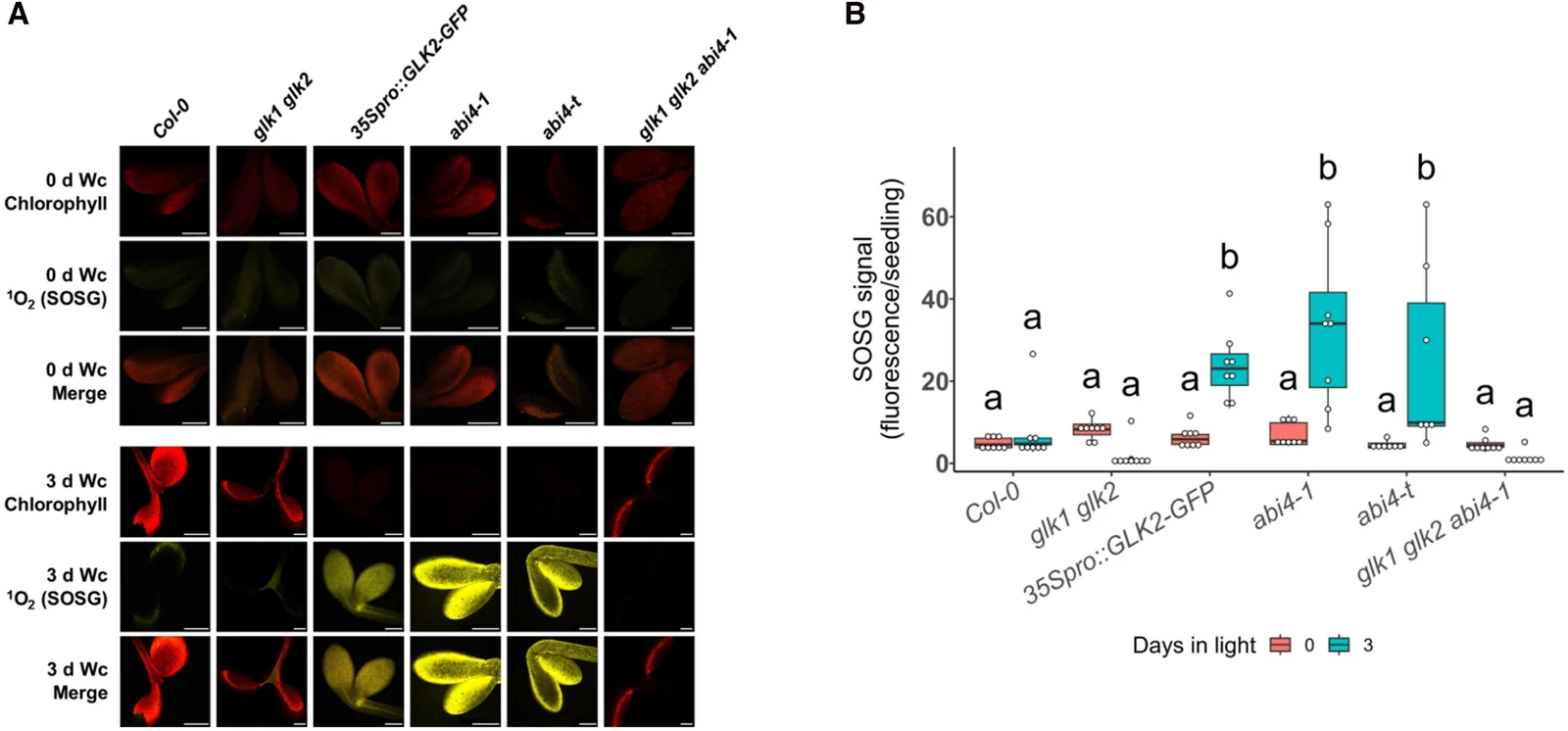
Photobleaching of etiolated, then light-exposed *abi4* mutant and *35Spro*::*GLK2*-*GFP* seedlings correlates with high singlet oxygen (^1^O_2_) accumulation. (A) Representative fluorescence microscopy images of chlorophyll autofluorescence and Singlet Oxygen Sensor Green (SOSG, detecting ^1^O_2_) fluorescence in 5-day-old etiolated seedlings before (0 d Wc) and after exposure to 100 µmol m^−2^ s^−1^ constant white light for 3 d (3 d Wc). Scale bars=200 µm. (B) Quantification of SOSG fluorescence per seedling in cotyledons of seedlings grown as in A. Groups which are significantly different are labeled with different letters (*n*=8 seedlings, ANOVA and Tukey’s post-hoc tests).

### Certain *PhANG* transcripts show GLK-dependent up-regulation in etiolated *abi4* mutant seedlings

We established above that the photobleaching of etiolated *abi4* mutant seedlings is likely to be due to an altered Pchlide homeostasis. Because *PhANG*s encode etioplast proteins responsible for Pchlide biosynthesis and photoreduction (Solymosi and Aronsson, 2013), and GLKs are transcriptional activators of *PhANG*s (Waters *et al*., 2009), we first hypothesized that *GLK* transcript levels may be altered in the *abi4* mutants. To test this, we quantified the transcript levels of *GLK1* and *GLK2* by performing RT–qPCR using RNA isolated from 5-day-old etiolated seedlings. However, as shown in Fig. 6A, both *abi4* mutants showed similar *GLK1* and *GLK2* transcript levels to those in the wild type, indicating that *ABI4* did not affect *GLK1* or *GLK2* transcript levels under these conditions. In the original publication that characterized the *glk1 glk2* mutant (Fitter *et al*., 2002), residual *GLK1* transcript was still detectable in the *glk1 glk2* mutant, probably because the *Atglk1-1* allele contains a dSpm insertion in the 5'-untranslated region (UTR) of *GLK1*. There were also no significant changes observed for *GLK1* nr *GLK2* transcript levels in the *glk1 glk2 abi4-1* mutant compared with the *glk1 glk2* mutant, indicating that *ABI4* did not affect *GLK1* or *GLK2* transcript levels in this genetic background either (Fig. 6A). Thus, the enhanced photobleaching observed in *abi4* mutants is not caused by a change in *GLK1* or *GLK2* transcript levels.

**Fig. 6.**
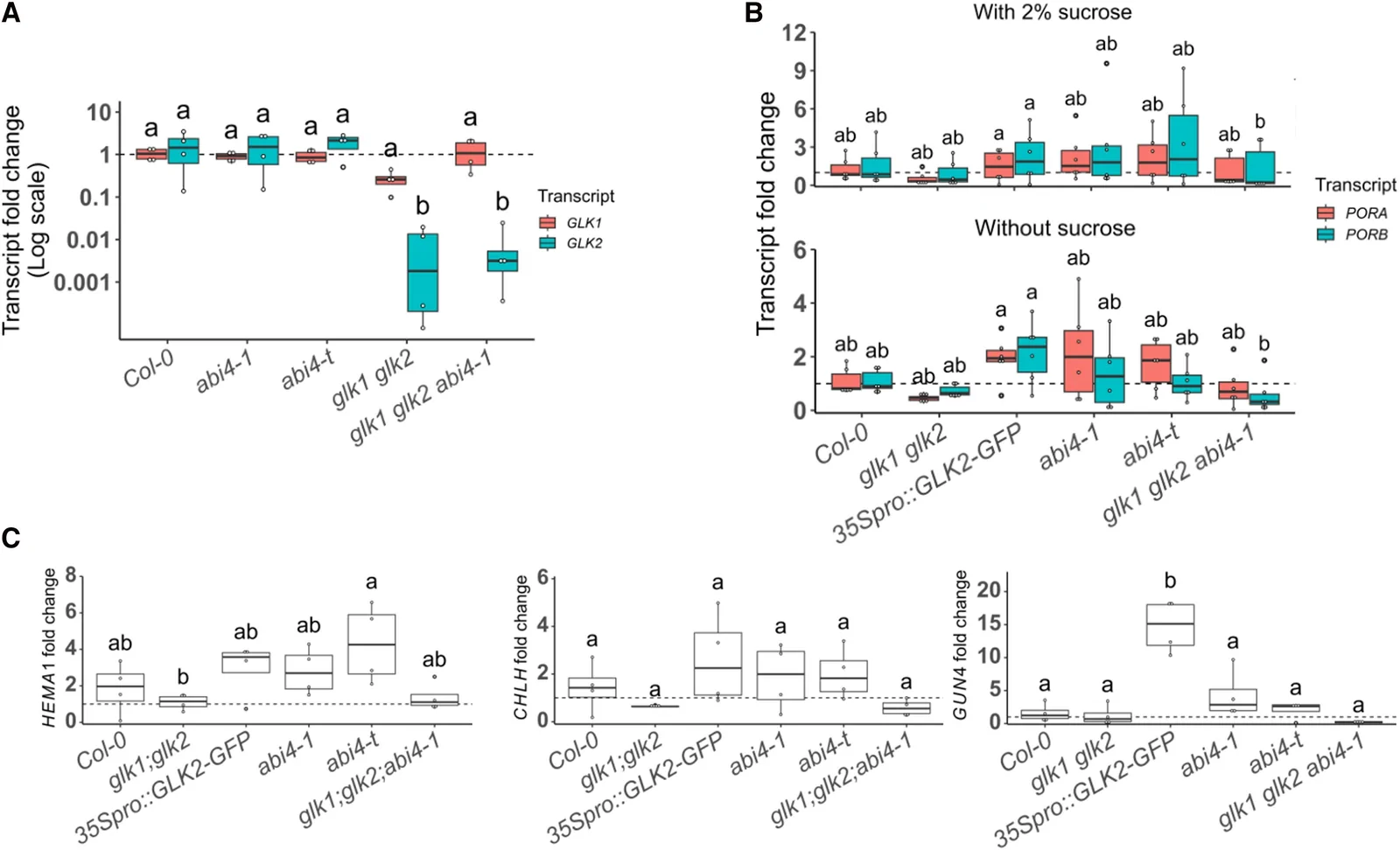
Transcript fold change of *GLK* genes and Pchlide biosynthesis genes in 5-day-old etiolated seedlings. Seedlings were grown on MS agar plates without sucrose (unless indicated otherwise), and the transcript levels were quantified using RT–qPCR. The mean transcript level in Col-0 was set to 1 (indicated by the dashed line), and data in each panel were compared with ANOVA and Tukey’s post-hoc tests. Genotypes which are significantly different are labeled with different letters. (A) Transcript levels of *GLK1* and *GLK2* are not altered in *abi4* mutants, *n*=4. (B) Transcript levels of *PORA* and *PORB* are not lower in *abi4* mutants with (top panel) or without 2% sucrose (bottom panel), *n*=6. (C) Three main Pchlide biosynthesis genes are targeted by GLKs (*n*=4 replicate extracts).

Next, we hypothesized that low *POR* transcript accumulation also contributed to the inefficient Pchlide photoreduction in *abi4* mutant seedlings. Since sucrose rescued the photobleaching phenotype (Fig. 4A) and sucrose indirectly promotes *POR* gene expression (Mu *et al*., 2022), we quantified the transcript levels of *PORA* and *PORB* in etiolated seedlings grown with or without 2% sucrose supplementation. As demonstrated in Fig. 6B, the transcript levels of *PORA* and *PORB* in the two *abi4* mutants were similar to those in the wild type, irrespective of the presence or absence of sucrose. In fact, the two *abi4* mutants resembled the *35Spro*::*GLK2*-*GFP* line in terms of having slightly higher transcript levels of both *POR* genes compared with those in the wild type. Therefore, we conclude that the photobleaching observed in etiolated *abi4* mutant seedlings is not caused by any change in *POR* transcript levels.

To investigate the cause of Pchlide overaccumulation in etiolated *abi4* mutants, we quantified the transcript levels of three main GLK target genes that are involved in Pchlide biosynthesis. As seen in Fig. 6C, both *HEMA1* and *CHLH* transcripts accumulated to slightly higher levels in the *35Spro*::*GLK2*-*GFP* line and the two *abi4* mutants compared with the wild type, although ANOVA and Tukey’s post-hoc tests did not indicate statistically significant differences. The *GUN4* transcript level was significantly up-regulated in the *35Spro*::*GLK2-GFP* line, and its transcript levels in the two *abi4* mutants were slightly elevated from the wild-type level, albeit not significantly. In comparison, the transcript levels of all three genes were lowest in *glk1 glk2* and *glk1 glk2 abi4*. Together, the data demonstrate that GLKs can at least partially promote the accumulation of *HEMA1*, *CHLH*, and *GUN4* transcripts in darkness, and the transcript accumulation patterns of these three genes may explain to a limited degree the Pchlide overaccumulation in etiolated *abi4* mutant seedlings.

An overexpression of *LHCB*, encoding the light-harvesting complex proteins surrounding PSII, has been reported to exacerbate cotyledon photobleaching (Liu *et al*., 2018). Since *LHC* genes are also a main class of GLK targets (Waters *et al*., 2009), we quantified their transcript levels in 5-day-old etiolated seedlings. Amongst the *LHC* genes we examined, five had a higher transcript level in the *35Spro*::*GLK2*-*GFP* line than in the wild type: *LHCB2.1*, *LHCB2.2*, *LHCB2.3*, *LHCB3*, and *LHCB6* (Fig. 7A). Their transcript levels were also either significantly or slightly elevated in the two *abi4* mutants. Importantly, the accumulation levels of these transcripts were again lowest in *glk1 glk2* and *glk1 glk2 abi4* mutants, showing that the high *LHC* induction in etiolated *abi4-1* mutant seedlings was completely dependent on *GLK* genes. Furthermore, because *LHCB* overexpression impairs cotyledon greening in the light by promoting precocious prothylakoid (PT) proliferation in darkness without affecting Pchlide accumulation (Liu *et al*., 2018), we examined the etioplast ultrastructure in these 5-day-old etiolated seedlings using TEM. As shown in Fig. 7B, we observed that the membrane structures within some etioplasts showed an ‘inverted staining’, where both the PLB and the PTs appeared as white instead of black against the dark stroma. The occurrence of such inverted staining did not strictly correlate to certain genotypes, since seedlings of the Col-0 and *abi4-t* genotypes contained both etioplasts that were stained normally and etioplasts with inverted staining. Nevertheless, the staining status *per se* did not affect the etioplast ultrastructure. When compared with the wild type, the *abi4* mutants and the *35Spro::GLK2-GFP* seedlings did not have significantly different etioplast sizes (Fig. 7C). However, the *abi4-t* mutant etioplasts showed significantly longer PT than the wild type, and this result remained the same when the PT length was adjusted to the total etioplast area (Fig. 7C). However, since only one of the two *abi4* mutants exhibited this phenotype, we conclude that the accumulation patterns of *LHCB* transcripts in darkness do not necessarily correlate with precocious etioplast-to-chloroplast differentiation.

**Fig. 7.**
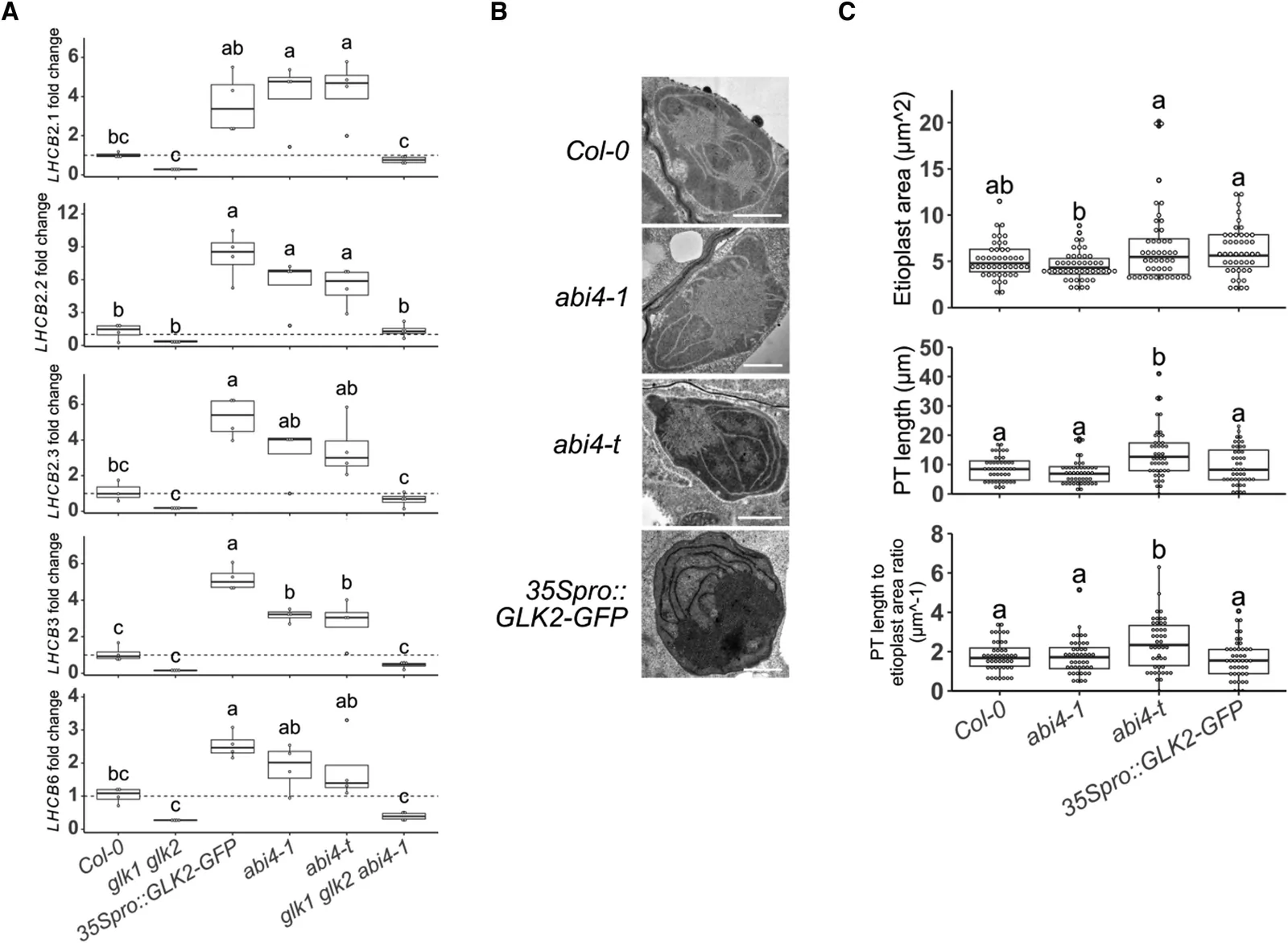
Seedlings of *abi4* mutants sometimes have overdifferentiated etioplasts. (A) Five *LHCB* genes show high transcript levels in etiolated *abi4* mutants seedlings. Seedlings were grown on MS agar plates in darkness for 5 d without sucrose, and the transcript levels were quantified using RT–qPCR. The mean transcript level in Col-0 was set to 1 (indicated by the dashed line), and data in each panel were compared with ANOVA and Tukey’s post-hoc tests. Genotypes which are significantly different are labeled with different letters (*n*=4 replicate extracts). (B) Representative images of the etioplast ultrastructure in 5-day-old etiolated cotyledons under TEM; scale bars=1 µm. (C) Quantification of etioplast area (upper panel), prothylakoid (PT) length (middle panel), and the PT length to etioplast area ratio (lower panel). Genotypes which are significantly different are labeled with different letters (ANOVA and Tukey’s post-hoc tests, *n*=45 etioplasts).

Lastly, since ABI4 physically interacts with GLK1 and GLK2, we hypothesized that ChIP may reveal certain regulatory regions of GLK target genes as physically interacting with ABI4 proteins. By examining previously published ChIP datasets acquired using transgenic Arabidopsis grown in various conditions (Waters *et al*., 2009; Reeves *et al*., 2011; Yu *et al*., 2016; Liu *et al*., 2020; Tu *et al*., 2022), we discovered that both ABI4 and either GLK1 or GLK2 bind to the regulatory regions of *LHCB2.2*, *LHCB2.4*, *LHCB4.2*, *LHCB6*, and *CHLH*. Together, these ChIP data corroborate the physical and functional ABI4–GLK interaction that we propose here.

## Discussion

In the present work, we identified ABI4 as a GLK-interacting protein in Y2H assay (Fig. 1) and *in planta* (Fig. 2). ABI4 belongs to the AP2/ERF transcription factor family and is well conserved in land plants (Gregorio *et al*., 2014). Arabidopsis loss-of-function *abi4* mutants were first identified based on their insensitivity to ABA during seed germination (Finkelstein, 1994; Finkelstein *et al*., 1998). ABI4 participates in additional signaling pathways, including responses to sugars and multiple hormones, while its role in retrograde signaling from the chloroplast to the nucleus has been controversial (Kacprzak *et al*., 2019; Chandrasekaran *et al*., 2020).

We report here that etiolated, dark-grown seedlings of two *abi4* mutants are phenotypically similar to seedlings overexpressing GLK2 (*35Spro*::*GLK2*-*GFP*): etiolated *abi4* mutant and *35Spro*::*GLK2*-*GFP* seedlings contain more Pchlide (Fig. 3), fail to green when exposed to light (Fig. 4), generate more ^1^O_2_ after the dark-to-light transition (Fig. 5), and accumulate higher transcript levels of *LHCB* genes in darkness (Fig. 7) than the wild type. This suggests that GLKs and ABI4 have antagonistic functions. Indeed, *glk1 glk2* mutant seedlings have opposite phenotypes to those of *abi4* mutants: etiolated *glk1 glk2* mutant seedlings contain less Pchlide (Fig. 3), green normally when exposed to light (Fig. 4), generate low ^1^O_2_ upon light exposure (Fig. 5), and accumulate slightly lower transcript levels of *PhANG*s in darkness (Figs 6, 7) than the wild type. The direct protein–protein interaction between GLKs and ABI4 indicates that the antagonistic activities of GLKs and ABI4 are a result of the GLK–ABI4 interaction. Our finding that the *abi4* mutant phenotype fully depends on *GLK1* and *GLK2* supports this idea and suggests that ABI4 acts upstream of GLK1 and GLK2 by inhibiting the activities of the GLK1 and GLK2 proteins. The alternative possibility that ABI4 represses the expression of *GLK1* and *GLK2* can be excluded since *GLK1* and *GLK2* transcript levels were unaltered in *abi4* mutants.

### GLK and ABI4 antagonistically impact cotyledon greening due to their effects on protochlorophyllide and singlet oxygen accumulation

In our greening assays, etiolated *abi4*-*1* and *abi4*-*t* mutant seedlings exhibited reduced greening rates compared with the wild type after the seedlings were exposed to light (Fig. 4). This is not due to any major defect in chlorophyll biosynthesis, because *abi4* mutant seedlings green normally when grown without prior etiolation (Fig.4C, Supplementary Figs S2, S4C) or even after 2 d of etiolation (Fig. 4A). This is confirmed by Shu *et al*. (2013) where both *abi4*-*1* and *abi4*-*t* mutant seedlings achieved similar greening percentages compared with Col-0 when grown with 1% sucrose and without prior etiolation. Since Pchlide is a photosensitizer that can inhibit seedling greening in light (Meskauskiene *et al*., 2001), we hypothesized that the photobleaching of etiolated *abi4* mutant seedlings is due to an overaccumulation of Pchlide in these seedlings. We found that etiolated *abi4* mutant seedlings, as well as etiolated *35Spro*::*GLK2*-*GFP* seedlings, accumulated more Pchlide than wild-type seedlings (Fig. 3), which correlated with lower greening rates after seedlings were exposed to light (Fig. 4). In contrast, the *glk1 glk2* mutant accumulated less total Pchlide than the wild type like etiolated seedlings (Fig. 3A, B; Supplementary Fig. S4A), and it showed high greening rates in the cotyledon greening assay (Fig. 4). Importantly, the *glk1 glk2* mutations returned both the Pchlide accumulation (Fig. 3) and the cotyledon greening rate (Fig. 4) of *abi4-1* mutant seedlings to a wild-type level. We therefore conclude that the Pchlide overaccumulation in etiolated *abi4* mutant seedlings causes the photobleaching of these seedlings after the dark-to-light transition, and this Pchlide overaccumulation may be due to de-repressed GLK activity.

Next, we hypothesized that the photobleaching of etiolated *abi4* mutant seedlings in light is caused by ^1^O_2_ generated by Pchlide photosensitization. ^1^O_2_ in plants is primarily generated in the plastids, either by chlorophyll molecules during photosynthesis or by chlorophyll precursors during dark-to-light transitions (Wang *et al*., 2020). ^1^O_2_ is toxic to the cell (Apel and Hirt, 2004), and its accumulation with other ROS species can lead to programmed cell death (Wagner *et al*., 2004; Kim *et al*., 2012). Because of the direct role of GLKs in promoting Pchlide biosynthesis, it was not surprising to find that etiolated *glk1 glk2* seedlings contained less ^1^O_2_ and showed a high greening rate, while etiolated *35Spro*::*GLK2-GFP* seedlings contained more ^1^O_2_ and showed a low greening rate upon light exposure compared with the wild type (Figs 4, 5). A recent publication also supports the conclusion that limiting GLK activity in dark-grown seedlings acts to promote cotyledon greening during de-etiolation (Tachibana *et al*., 2025). We also observed high ^1^O_2_ levels and low greening rates in both *abi4* mutants after their etiolated seedlings were transferred to light (Figs 4, 5). This seems to contrast with a previous study in which two *abi4* loss-of-function mutations both rescued the lethal phenotype of the *flu* mutant under dark/light cycles (Zhang *et al*., 2023). The *flu* mutant seedlings accumulate at least four times as much Pchlide as the wild type during etiolation, leading to rapid cotyledon bleaching and death after the dark-to-light transition (Meskauskiene *et al*., 2001; Zhang *et al*., 2023). Intriguingly, the *abi4* mutations did not alter the Pchlide accumulation in etiolated *flu* mutant seedlings, or the ^1^O_2_ accumulation upon light exposure, but they lowered the transcript levels of ^1^O_2_-responsive genes in light (Zhang *et al*., 2023). Since chloroplast ^1^O_2_ stress responses are controlled by multiple pathways (Tano *et al*., 2023), we suggest that the pathways mediating the photobleaching of etiolated *flu* and *abi4* mutant seedlings in light may be different.

We further hypothesized that etiolated *abi4* mutant seedlings accumulate high levels of Pchlide due to an up-regulated Pchlide biosynthetic pathway. We showed that the *HEMA1*, *CHLH*, and *GUN4* transcript levels in the etiolated seedlings of the *35Spro*::*GLK2*-*GFP* line and the two *abi4* mutants were at least slightly elevated compared with the wild type (Fig. 6C). These transcripts possibly up-regulated the Pchlide biosynthesis in the seedlings. The similarity between etiolated *35Spro*::*GLK2*-*GFP* and *abi4* mutant seedlings suggests again that GLK protein activity may be de-repressed in etiolated *abi4* mutant seedlings. *HEMA1*, *CHLH*, and *GUN4* are activated by GLKs in light-grown plants (Waters *et al*., 2009), and we demonstrated here that their transcript accumulation in etiolated seedlings in the *abi4-1* mutant background is dependent on *GLK1* and *GLK2* (Fig. 6C). As expected, these *GLK*-dependent transcript levels correlated with the *GLK*-dependent Pchlide accumulation during etiolation (Figs 3A, B; Supplementary Fig. S3A) and greening patterns after the dark-to-light transition (Fig. 4). Since the transcript levels presented here came from end-point analyses, future studies can generate transgenic Arabidopsis lines each expressing a luciferase driven by promoters of *HEMA1*, *CHLH*, or *GUN4*, whereby the transcriptional dynamics of these promoters can be monitored in real-time by luciferase luminescence during etiolation and compared among different genotypes. Additional GLK and ABI4 target genes should be identified in order to fully explain the Pchlide accumulation pattern observed here.

We also investigated whether the *POR* transcript level is altered in etiolated *abi4* mutant seedlings, due to the direct role of PORs in Pchlide photoreduction. Knocking out either *PORA* or *PORB* produces seedlings with smaller PLBs, unstructured PTs, and a delayed-greening phenotype (Masuda *et al*., 2003, 2009). *PORA* and *PORB* are both transcriptionally activated by GLKs in seedlings grown under a long-day photoperiod (Waters *et al*., 2009). In comparison, we observed only a slight transcript overaccumulation of either *POR* gene in etiolated *35Spro*::*GLK2*-*GFP* seedlings compared with the wild type (Fig. 6B). Moreover, we observed no significant change in *PORA* and *PORB* transcript levels in both *abi4* mutants compared with the wild-type etiolated seedlings (Fig. 6B). This differs from the report of Xu *et al*. (2016), where etiolated *abi4-1* mutant seedlings were shown to contain a lower *PORA* transcript level compared with that in the wild type. Our observation remained the same, either without supplementary sucrose in the growth medium, or with 2% supplementary sucrose, as used by Xu *et al*. (2016). We therefore conclude that the *POR* transcript level is unlikely to have significantly affected our results.

### Impact of GLKs and ABI4 on the expression of *PhANG* genes and on the etioplast structure

Beyond the PLB, the ultrastructure of the PTs also affects the greening process (Liu *et al*., 2018; Li *et al*., 2024). Overexpressing either *LHCB1.1* or *LHCB2.1* produces seedlings with low greening rates when the seedlings are exposed to light after prolonged etiolation (Liu *et al*., 2018). These transgenic seedlings contain a wild-type level of total Pchlide, but they develop etioplasts with no PLBs and only overproliferated PTs. This agrees with our findings that the *35Spro*::*GLK2*-*GFP* line and the two *abi4* mutants, which accumulated more *LHCB* transcripts than the wild type as etiolated seedlings (Fig. 7A), showed lower greening rates in the cotyledon greening assay (Fig. 4). In seedlings of these genotypes, only the *abi4-t* mutant exhibited overproliferated PTs compared with the wild type (Fig. 7B, C). This could be due to the fact that TEM sections only represent the three-dimensional etioplast ultrastructure at a two-dimensional level, and that etioplasts show high structural variation. Amongst the different LHC isoforms, at least LHCB2 accumulates during seedling etiolation to a level detectable by western blotting (Armarego-Marriott *et al*., 2019), therefore, future studies can investigate whether the *35Spro*::*GLK2*-*GFP* line and the two *abi4* mutants accumulate more LHCB proteins than the wild type in etiolated seedlings. Additionally, we observed that some etioplasts showed an ‘inverted staining’ (Fig. 7B). Although such inverted staining has occurred in other etioplast studies before (Vinti *et al*., 2005; Liang *et al*., 2022), we presently cannot explain why in our experiment even seedlings of the same genotype were found to contain both normally stained and invertedly stained etioplasts.

### Model on the antagonistic activities of GLKs and ABI4

The present study suggests that the ABI4–GLK protein–protein interaction regulates the greening of etiolated seedlings in light, and this agrees with previous reports where other protein–protein interactions between nuclear proteins regulate Pchlide biosynthesis and etioplast development (Chen *et al*., 2013; Liu *et al*., 2018; Sun *et al*., 2019). At present, the mechanism(s) by which ABI4 represses GLK activity is not clear and, in addition to darkness, this repression could occur in other conditions where limiting *PhANG* expression becomes necessary. ABI4 may reduce GLK protein stability, inhibit GLK dimerization, limit GLK binding to its target promoters, and/or repress the transcriptional activation capacity of GLK. D. Zhang *et al*. (2021) reported that GLK1 and GLK2 are protected from proteasomal degradation after being phosphorylated by BRASSINOSTEROID INSENSITIVE 2 (BIN2) and, since BIN2 is inactivated by darkness, GLK proteins are less stable in darkness. N. Kim *et al*. (2023) revealed that REPRESSOR OF PHOTOSYNTHETIC GENES 1 (RPGE1) represses GLK1 homodimerization and DNA binding, thereby limiting *PhANG* activation in shade. Intriguingly, it was shown by M. Li *et al*. (2022) that treating plants with salicylic acid alters the GLK interactome, reduces the binding of GLK to *PhANG* promoters, and enhances the stress response. These reports illustrate the various mechanisms whereby GLK protein activity can be modulated.

Since ABI4 physically interacts with GLK, a direct binding of ABI4 to GLK target promoters is not necessary for ABI4 to inhibit GLK target gene expression. Nevertheless, ABI4 has been shown to bind *LHCB* promoters in Y1H assay, *in vitro* and *in planta* (Koussevitzky *et al*., 2007; Guo *et al*., 2016; Zhang *et al*., 2023). We therefore cannot exclude the possibility that ABI4 can regulate the expression of certain *PhANG*s independently of GLKs, or that ABI4 regulates GLK functions in light, similar to how PSEUDO-ETIOLATION IN LIGHT (PEL) proteins suppress GLK activities (Han *et al*., 2024). Nonetheless, the antagonistic GLK–ABI4 interaction that we propose here may integrate another layer of complexity into a transcriptional factor network based on direct protein–protein interactions, including those between GLKs and HY5 (Zhang *et al*., 2024) and those between ABI4 and PIF4 (Luo *et al*., 2024; Song *et al*., 2024). The co-action of these transcriptional factors would serve to fine-tune the pigment biosynthesis and plastid development according to the needs of the developing seedling.

## Supplementary Material

erag364_Supplementary_Data

## Data Availability

The primary data supporting this study were not made publicly available at the time of publication. Data will be made available upon request to the corresponding authors, PY or UH.

## Supplementary data

The following supplementary data are available at *JXB* online.

Fig. S1. CT values of *ACT2* remained stable in RT–qPCR experiments.

Fig. S2. Chlorophyll accumulation is not altered in light-grown *abi4* mutants.

Fig. S3. Verification of the *glk1 glk2 abi4-1* triple mutant.

Fig. S4. Replicate experiments of Pchlide and chlorophyll quantification.

Table S1. Primers used in this study.
