# Supplementary material for: Arabidopsis GOLDEN2-LIKE transcription factors interact with ABA-INSENSITIVE 4 to modulate cotyledon greening in light-exposed etiolated seedlings
Source: J Exp Bot. 2026 Aug 4;77(19):6792–808. doi: 10.1093/jxb/erag364 (PMC13672994; doi:10.1093/jxb/erag364)
Supplement: Supplementary file 1 [file erag364_supplementary_data.pdf]

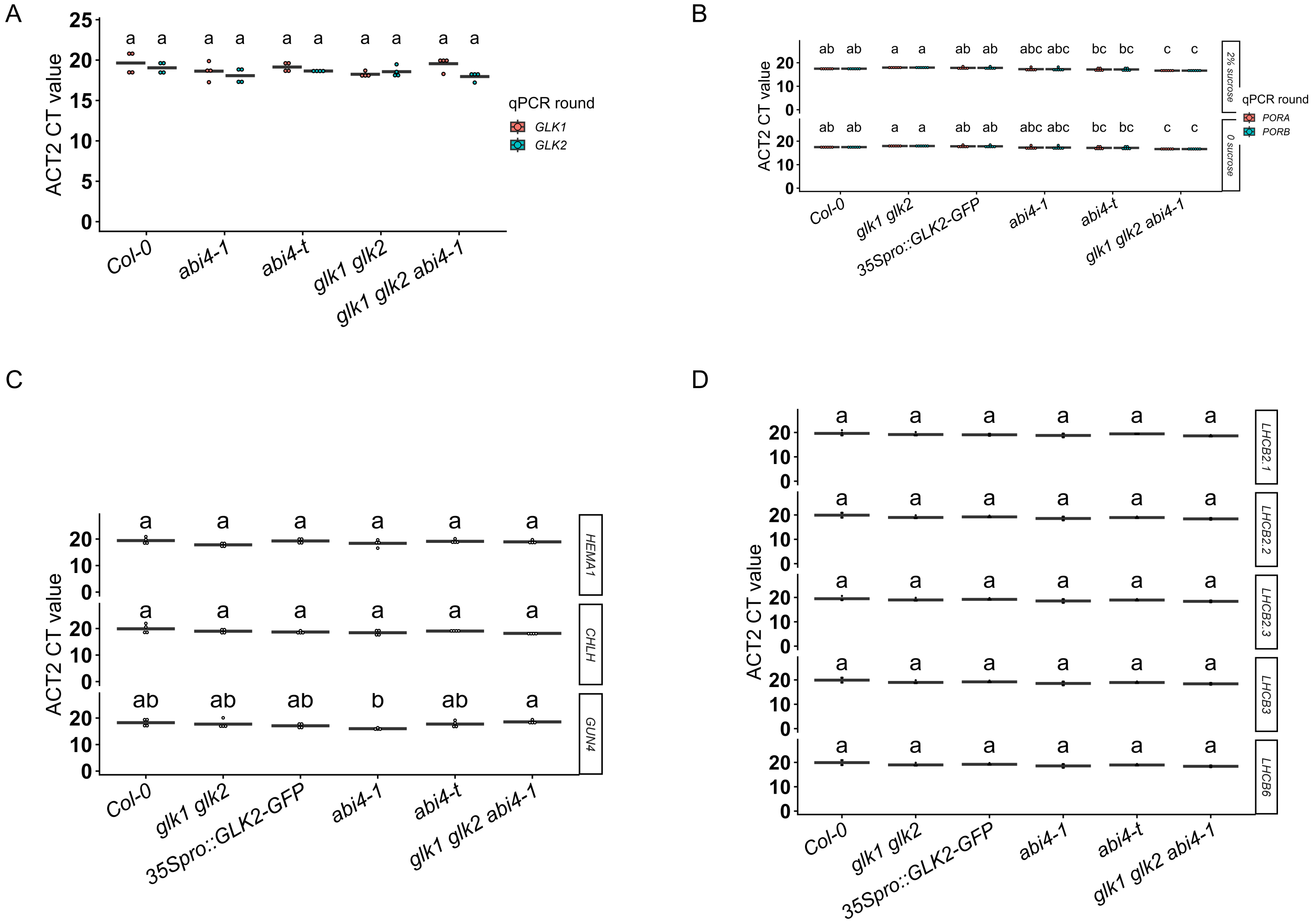

Figure S1. CT values of *ACT2* remained stable in RT-qPCR experiments.
A corresponds to Fig. 6A. B corresponds to Fig. 6B. C corresponds to Fig. 6C.
D corresponds to Fig. 7A.

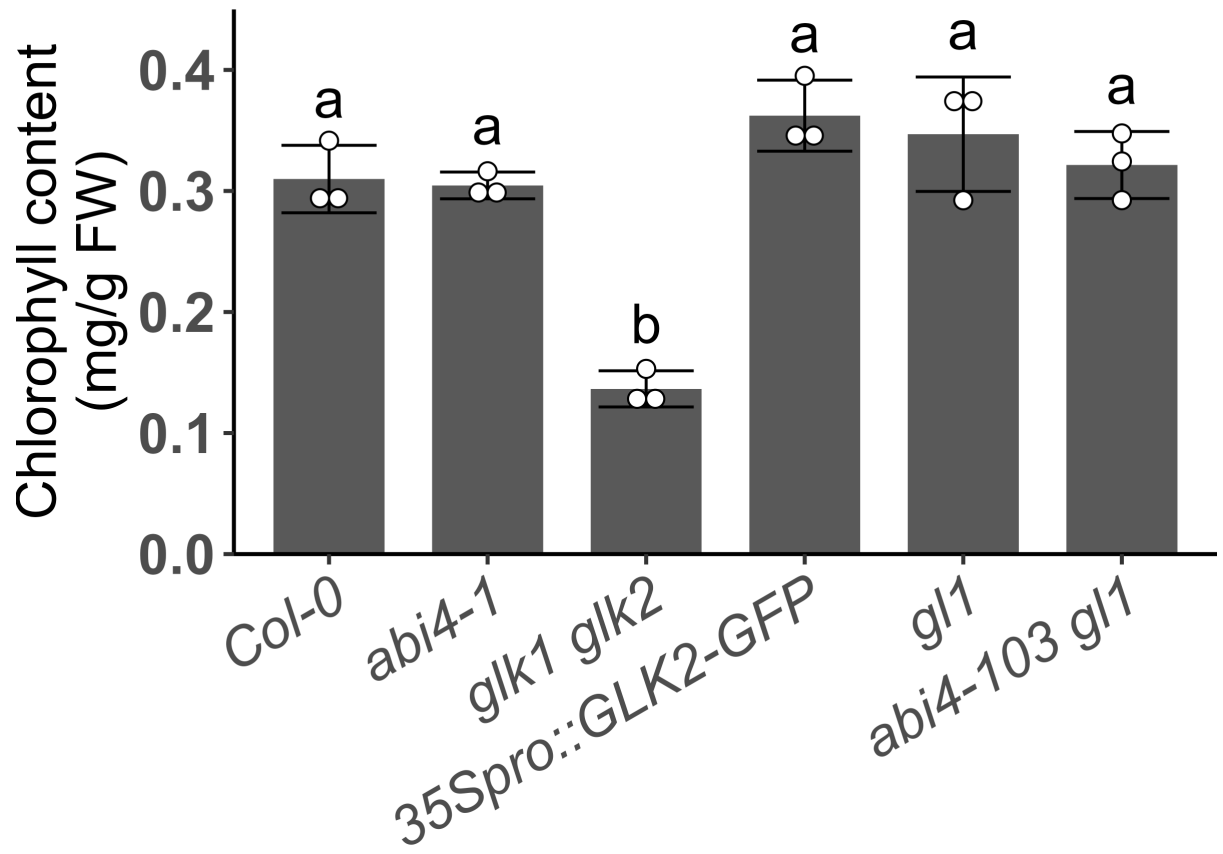

Figure S2. Chlorophyll accumulation is not altered in light-grown *abi4* mutants. Seedlings are grown for 10 days on soil in a long-day phytochamber with 100 μmol photons m^{-2} s^{-1} white light. Data were normalized to per gram fresh weight (/g FW). Genotypes which are significantly different are labelled with different letters (n=3 replicate extracts, ANOVA and Tukey's post hoc tests), error bars indicate SD.

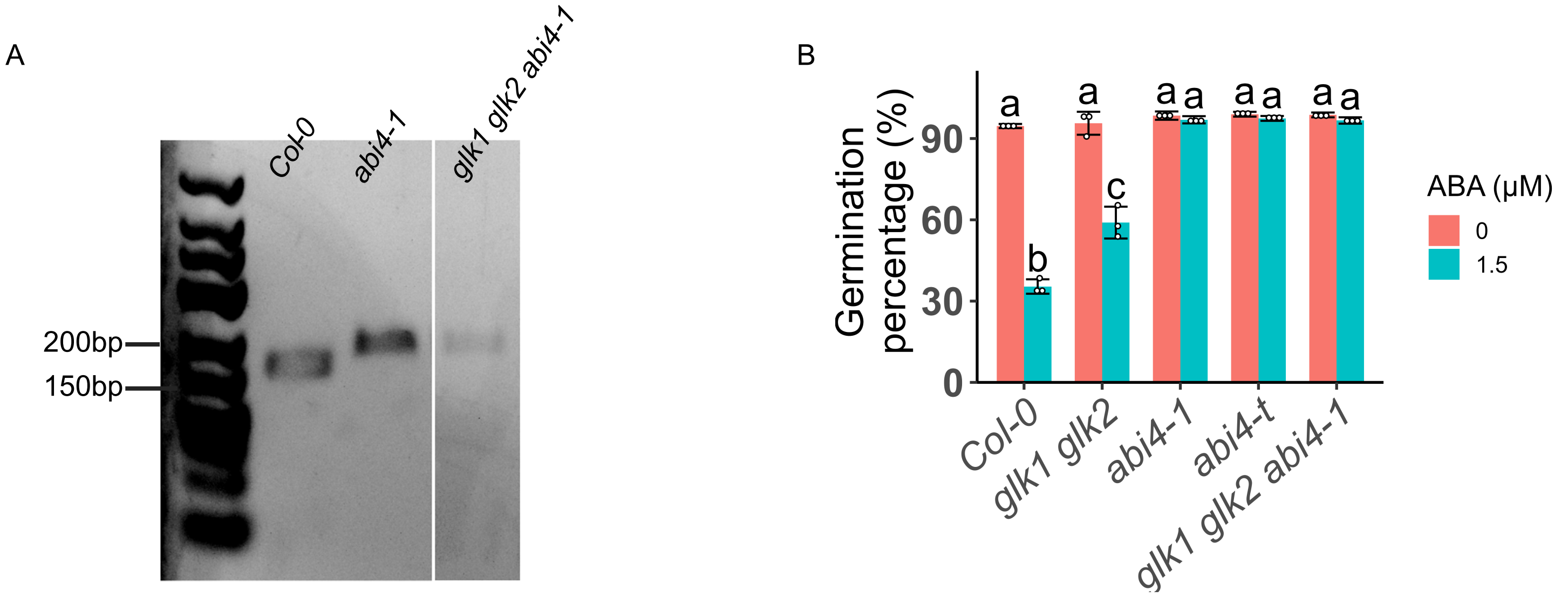

Figure S3. Verification of the *glk1 glk2 abi4-1* triple mutant. A, Genotyping by dCAPS for detecting homozygous *abi4-1*, samples were ran on a 3% agarose gel at 100 V for 25 min. GeneRuler low range DNA ladder was used in the left most lane. The *Col-0* fragment is predicted to be 25 bp smaller than the *abi4-1* fragment. B, Germination phenotype on MS agar plates ± ABA. Genotypes which are significantly different are labelled with different letters (n=3 replicate plates, ANOVA and Tukey's post hoc tests), error bars indicate SD.

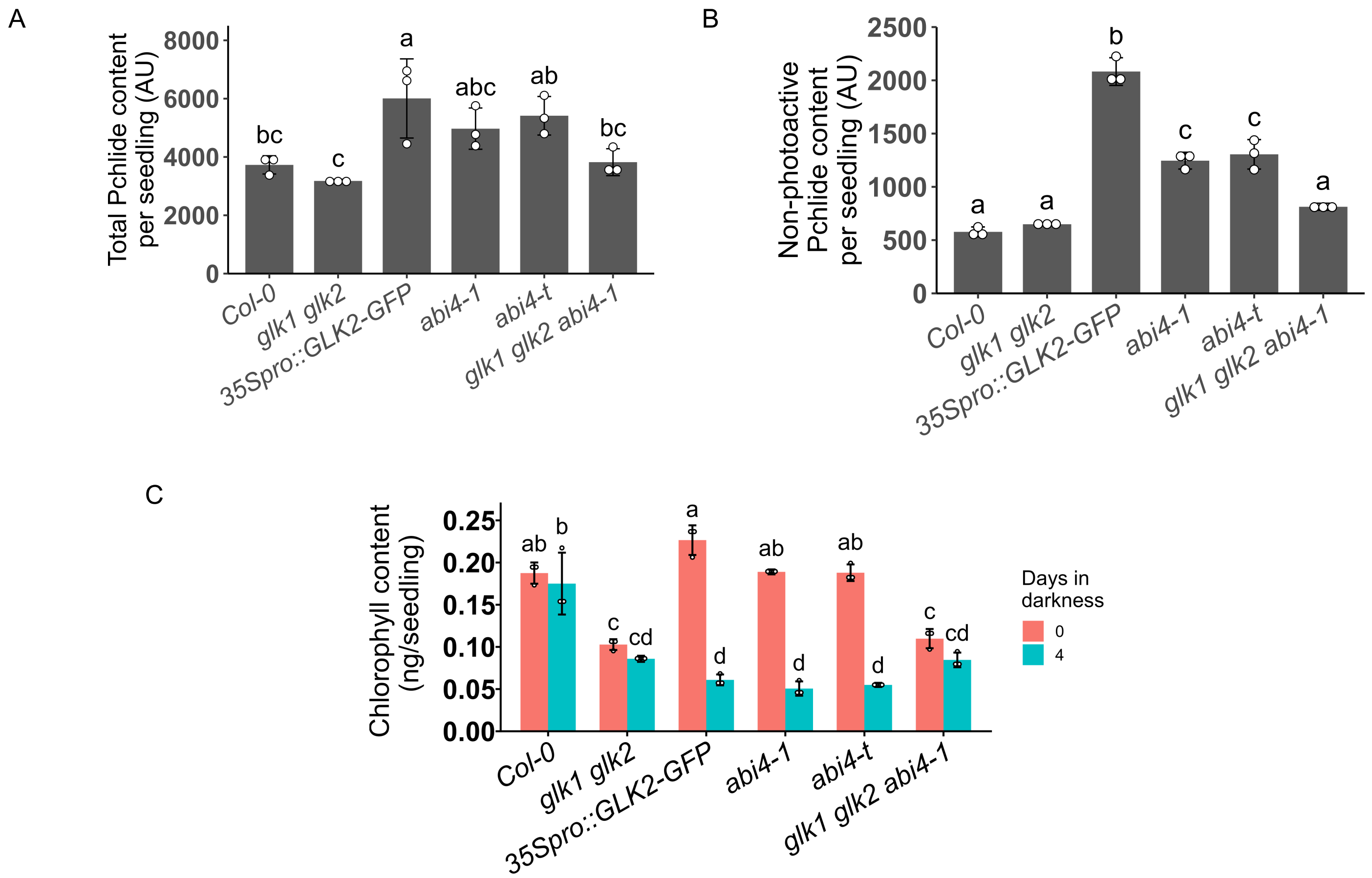

Figure S4. Replicate experiments of Pchlide and chlorophyll quantification. A corresponds to Fig. 3B. B corresponds to Fig. 3. C corresponds to Fig. 4c.

Supplementary Table S1. Primers used in this study

Primers used for dCAPS	
abi4-1-genotyping-AluI-F	CTCAACTTAACCCCTTCGTCTCCTTC
abi4-1-genotyping-AluI-R	GGGATACCGTACGGACCAAAGTTAG
Primers used for cloning Y2H constructs	
pENTR3C-GLK1-F	ggatccggtaccgaattcATGTTAGCTCTGTCTCCGGC
pENTR3C-GLK1-R	gagtgcggccgcgaattcTCAGGCACAAGACGCGG
pENTR3C-GLK2-F	ggatccggtaccgaattcATGTTAACTGTTTCTCCGGCTC
pENTR3C-GLK2-R	gagtgcggccgcgaattcTCAAGGAAGAGGAGGAACAT
pENTR3C-GLK1$_{\Delta 1\text{-}154}$-F	ACTGGATCCGGTACCGAATTcAAAGTGGATTGGACACCAGAGC
pENTR3C-GLK1$_{\Delta 1\text{-}154}$-R	CTCGAGTGCGGCCGCGAATTTCAGGCACAAGACGCGGTCG
pENTR3C-GLK2$_{\Delta 1\text{-}148}$-F	ACTGGATCCGGTACCGAATTCAAGGTGGATTGGACGCCGGA
pENTR3C-GLK2$_{\Delta 1\text{-}148}$-R	CTCGAGTGCGGCCGCGAATTTCAAGGAAGAGGAGGAACATTAGAAA
pENTR3C-ABI4$_{\Delta 188\text{-}289}$-F	ACTGGATCCGGTACCGAATTcATGGACCCTTTAGCTTCCCA
pENTR3C-ABI4$_{\Delta 188\text{-}289}$-R	CTCGAGTGCGGCCGCGAATTATAGAATTCCCCCAAGATGGGA
Primers used for cloning LCI constructs	
pCAMBIA1300-GLK1-nLUC-F	gacgagctcggtaccATGTTAGCTCTGTCTCCGGCG
pCAMBIA1300-GLK1-nLUC-R	gtacgagatctggtcgacGGCACAAGACGCGGTCG

pCAMBIA1300-GLK2-nLUC-F	ggggacgagctcggtaccATGTTAACTGTTTCTCCGGCTCCA
pCAMBIA1300-GLK2-nLUC-R	gcgtacgagatctggtcgacAGGAAGAGGAGGAACATTAGAAACTCC
pCambia1300-cLUC-ABI4-F	tacgcgtcccggggcggtaccATGGACCCTTTAGCTTCCCA
pCambia1300-cLUC-ABI4-R	acgaaagctctgcaggtcgacTTAATAGAATTCCCCCAAGATGG
Primers used for qPCR	
GLK1-F	CCGTATTTACCGACCGTAGCTACGAGA
GLK1-R	TACATCGTGTGATGCGGCGGCAGAG
GLK2-F	TGTGTGTAAGCAAGAGGGTGG
GLK2-R	CTACCCCTAATTGCTCCACCG
PORA-F	CCCTCTTCCCTCCTTTCCAG
PORA-R	GCTCCAATACACTCCCGACTTC
PORB-F	ACTTGCTCAGGTGGTGAGTG
PORB-R	CCACACTTTACGAGCCTTCT
HEMA1-F	GCTTCTTCTGATTCTGCGTC
HEMA1-R	GCTGTGTGAATACTAAGTCCAATC
CHLH-F	CATTGCTGACACTACAACTGC
CHLH-R	CTTCTCTATCTCACGAACTCCTTC
GUN4-F	CAATCTCACTTCGGACCAAC
GUN4-R	TTGAAACGGCAGATACGG
LHCB2.1-F	ACCCGGAGACATTCGCTAAGAAC
LHCB2.1-R	TGGATCAAGTTAGGGTTTCCGAGG
LHCB2.2-F	AGCAGATGGGCTATGTTGGGT
LHCB2.2-R	CTCAGCCAAGTTCAATGGGTCG
LHCB2.3-F	CGTCAAGTCTACTCCTCAGAGCATC
LHCB2.3-R	TAATATGCTTTGCGCGTGGATCAAG
LHCB3-F	GCCGGTTCACAAATCTTCTCCG
LHCB3-R	AGTAACTGGATCATCAGCGAGACC
LHCB6-F	TGGCTCGCTACCAGGAGATTTC
LHCB6-R	CAATTGGGTTCCAAGAAGCGACC
RD29B-F	CAAAGCGAGAGCCCTGTAAAA
RD29B-R	CGTAAACCGGAGTCAACTTCTCA
